# Urinary miRNA Analysis for Clear Cell Renal Cell Carcinoma: *miR-20a* as a Key Endogenous Normalizer

**DOI:** 10.3390/ijms27073323

**Published:** 2026-04-07

**Authors:** Giovanni Cochetti, Giacomo Vannuccini, Matteo Mearini, Alessio Paladini, Francesca Cocci, Raffaele La Mura, Daniele Mirra, Giuseppe Giardino, Ettore Mearini

**Affiliations:** Urology Clinic, Department of Medicine and Surgery, Santa Maria della Misericordia Hospital, University of Perugia, 06129 Perugia, Italy; giovanni.cochetti@unipg.it (G.C.); alessiopaladini89@gmail.com (A.P.); francesca.cocci@specializzandi.unipg.it (F.C.); raffaele.lamura94@gmail.com (R.L.M.); daniele.mirra@specializzandi.unipg.it (D.M.); giuseppe.giardino@specializzandi.unipg.it (G.G.); ettore.mearini@unipg.it (E.M.)

**Keywords:** urinary microRNAs (miRNAs), clear cell renal cell carcinoma (ccRCC), endogenous miRNA normalizer, *miR-20a*, liquid biopsy, normalization strategies

## Abstract

Urinary microRNAs (miRNAs) are promising noninvasive biomarkers for cancer detection, but their clinical utility is reduced by inconsistent normalization strategies, reducing reproducibility and comparability across studies. In this study, we assessed the stability of *miR-20a* as an endogenous normalizer for urinary miRNA profiling in clear cell renal cell carcinoma (ccRCC) while standardizing the pre-analytical phase using a urine stabilizing solution. Ninety-nine urine samples were analyzed: 47 from healthy individuals, 30 from ccRCC patients pre-surgery, and 22 post-operative patients. Six candidate miRNAs—*miR-20a*, *miR-15b*, *miR-16*, *miR-15a*, *miR-210-3p*, and *miR-let-7b*—were quantified via RT-qPCR. Stability analysis with RefFinder, integrating multiple algorithms (geNorm, normFinder, BestKeeper, and ΔCt methods), identified *miR-20a* as the most stable among the six candidates. Raw Ct values of *miR-20a* were normally distributed (Shapiro–Wilk test, *p* > 0.05), with no significant intergroup differences (one-way ANOVA, F(2.96) = 2.324, *p* = 0.103) and minimal intragroup variability (CV% 4.98–6.38). *MiR-20a* expression remained stable across different tumor staging, grading, and urine storage durations. These findings confirm miR-20a as a robust endogenous normalizer for urinary miRNA analyses and support the feasibility of developing reproducible urinary liquid biopsy workflows for ccRCC, even in settings where immediate sample processing is not feasible.

## 1. Introduction

Renal cell carcinoma (RCC) accounts for approximately 2% of all solid malignancies, with a relatively high incidence reported in Western countries. In 2022, approximately 434,840 new cases of RCC were diagnosed worldwide, with a mortality of approximately 155,953 [1]. Clear cell renal cell carcinoma (ccRCC) represents the most prevalent histological subtype, accounting for 65–70% of cases [2]. Because RCC is an asymptomatic tumor in the early stages, it often progresses undetected, leading to advanced disease, thus resulting in poor survival outcomes [3]. Currently, despite advancements in imaging techniques, approximately 60% of RCC cases are incidentally discovered, while nearly 20% are already metastatic at the time of diagnosis. These findings highlight the need to investigate new and accurate tools that not only allow the diagnosis of RCC but also provide information on the biology and prognosis of the disease. MicroRNAs (miRNAs) have emerged as promising biomarkers for cancer diagnosis, prognosis, and treatment response assessment. MiRNAs are a class of small, noncoding, single-stranded RNAs with an average length of 18–22 nucleotides that regulate gene expression at the post-transcriptional level by inhibiting the translation or promoting the degradation of target mRNAs [4]. MiRNAs play a key role in the control of homeostasis, regulating approximately 10–30% of all human genes [5]. One of the main features of miRNAs is their remarkable stability in biological fluids such as urine, plasma, and serum [6], which makes them excellent options for developing non-invasive biomarkers [7,8,9] applicable in liquid biopsy settings. Accurate quantification of miRNAs by Real-Time quantitative PCR (RT-qPCR) in urine samples is crucial for the development of reliable diagnostic assays. In this field, normalization strategies remain a major challenge due to the lack of universally accepted endogenous controls that are stably expressed across different pathological conditions [10,11]. An ideal endogenous normalizer should be consistently expressed in both healthy individuals and patients, regardless of disease status or treatment [12]. Additionally, endogenous normalizers allow for the correction of intrasample and intersample variability that may arise from differences in RNA input amounts, sample handling, or storage conditions [12]. J. Oto et al. (2021) [13] were the first to demonstrate the effectiveness of miR-20a-5p as a urinary endogenous normalizer in ccRCC, showing its stability and reproducibility across urine samples. In this context, the present work aimed to further validate miR-20a as an endogenous urinary normalizer, in order to match the findings of J. Oto et al. [13], in a larger cohort and in stabilized urine samples, as well as its consistency across relevant clinicopathological parameters including tumor grade and stage. Importantly, urine stabilization prevents genetic material degradation at room temperature for up to two years, overcoming the limitations associated with delayed sample processing. This approach facilitates sample storage and transport from peripheral outpatient settings lacking immediate laboratory facilities, thereby making urinary miRNA analysis more feasible and suitable for real-world clinical practice outside centralized laboratory environments.

## 2. Results

### 2.1. Baseline Demographics, Sample Characteristics, and Technical Quality Controls Using Synthetic Spike-In RNAs

Before analyzing miRNA expression patterns, the age and sex distribution of the population was first examined to assess potential sources of bias. No significant differences were found in the sex distribution (χ^2^ (2) = 2.773, *p* = 0.250) or age (Kruskal–Wallis H(2) = 0.064, *p* = 0.969). The median time from urine collection to RNA extraction was 21.8 days (IQR: 0–9 days) for the healthy group, 107.7 days (IQR: 54–119 days) for the ccRCC pre-surgery group, and 92.9 days (IQR: 43–119 days) for the ccRCC post-surgery patients. All the results of the above-described statistical tests are listed in Table 1.

**Figure 1:**
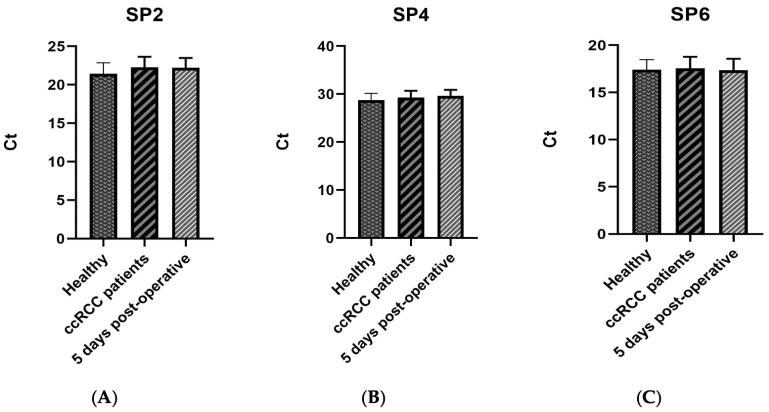
Comparison of Raw Ct values for three synthetic spike-in RNAs across clinical groups: healthy individuals, ccRCC patients, and 5 days post-operative samples. (**A**) Raw Ct values for Spike-in 2 and (**B**) Spike-in 4, used to assess the consistency of RNA extraction across different clinical groups. (**C**) Spike-in 6 was included to evaluate reverse transcription efficiency. Data are shown as mean ± standard deviation (SD) within each group.

To ensure that RT-qPCR miRNA quantification was not affected by technical or interindividual variability, synthetic nonhuman spike-in RNAs (Qiagen ©, Hulsterweg 82, 5912 PL Venlo, The Netherlands) were employed throughout the workflow, according to the manufacturer’s instructions. Specifically, *Spike-in 2* and *Spike-in 4* RNAs were added during all RNA isolation steps to monitor extraction efficiency, whereas *Spike-in 6* RNA was included in every reverse transcription reaction to assess the retro-transcription performance. No significant differences in the Ct values of these spike-ins were observed among the study groups (Figure 1), confirming consistent and efficient RNA extraction and reverse transcription across all of the samples.

### 2.2. Evaluating the Best MiRNA Normalizers

To identify the most stable reference gene among the six candidate miRNAs (*miR-15b*, *miR-16*, *miR-15a*, *miR-210-3p*, *miR-let-7b*, and *miR-20a-5p*), we used the RefFinder online tool (https://www.ciidirsinaloa.com.mx/RefFinder-master/?type=reference, accessed on 20 January 2025) [14]. *miR-20a* emerged as the top reference in Normfinder, BestKeeper, *miR-15b* ranked as the top reference in the Delta CT method, while the *miR-15b–miR-16* pair showed the highest stability according to Genorm. Conversely, *miR-210-3p* exhibited the poorest stability in all methods. Detailed results are reported in Figure S1 in the Supplementary Materials.

**Figure 2:**
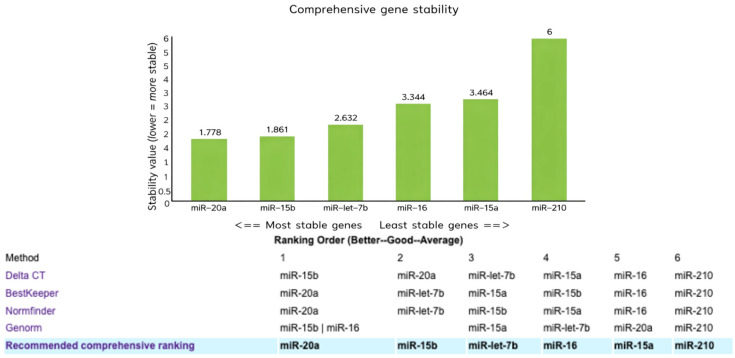
Comprehensive stability ranking of candidate reference miRNAs on the basis of the geometric mean of their rankings across multiple algorithms (Delta CT, BestKeeper, NormFinder, GeNorm). *MiR-20a* and *miR-15b* displayed the highest stability (geometric mean = 1.778 and 1.861, respectively), whereas *miR-210-3p* was the least stable (geometric mean = 6.000). The lower table shows the detailed rankings by each algorithm.

According to the comprehensive stability assessment provided by RefFinder, which combined the results of the 4 independent algorithms Delta CT, BestKeeper, Normfinder, and Genorm, *miR-20a* exhibited the highest stability, with a geometric mean (GM) ranking value of 1.778, followed closely by *miR-15b* (GM = 1.861). *MiR-let-7b* displayed intermediate stability (GM = 2.632), whereas *miR-16* and *miR-15a* were less stable, with GM values of 3.344 and 3.464, respectively. *MiR-210-3p* showed the lowest stability among the candidates, with a GM value of 6.000 (Figure 2).

After confirming with the online tool RefFinder that *miR-20a* was the most stable reference miRNA, descriptive statistics were used to summarize the distribution of the variable across the three clinical groups under study: healthy controls, ccRCC patients preoperative, and ccRCC patients at postoperative day 5. Data are reported as the mean ± standard deviation (SD), standard error of the mean (SEM), and 95% confidence intervals (95% CI), together with the minimum and maximum values (Table 2).

The Shapiro–Wilk test showed no significant deviation from normality in any group (W = 0.963, *p* values = 0.140 for the healthy controls, W = 0.959, *p* = 0.288 for the ccRCC preoperative patients, W = 0.983, *p* = 0.954 for postoperative samples) (Table 3). This validates the use of parametric statistical analyses like ANOVA for group comparisons.

**Figure 3:**
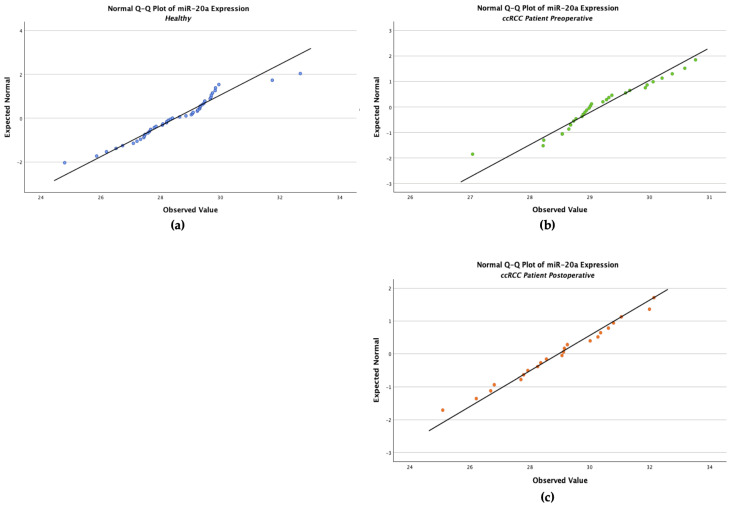
Normal Q–Q plots evaluating the distribution of *miR-20a* expression across different clinical groups: (**a**) healthy controls, (**b**) patients with clear cell renal cell carcinoma preoperative, and (**c**) patients 5 days post-nephrectomy.

In the normal Q–Q plots of all three clinical groups (Figure 3), the observed values closely aligned with the expected normal distribution line, suggesting that *miR-20a* expression follows an approximately normal distribution. The slight departures at the tails were within the acceptable range and do not indicate significant deviations from normality.

**Figure 4:**
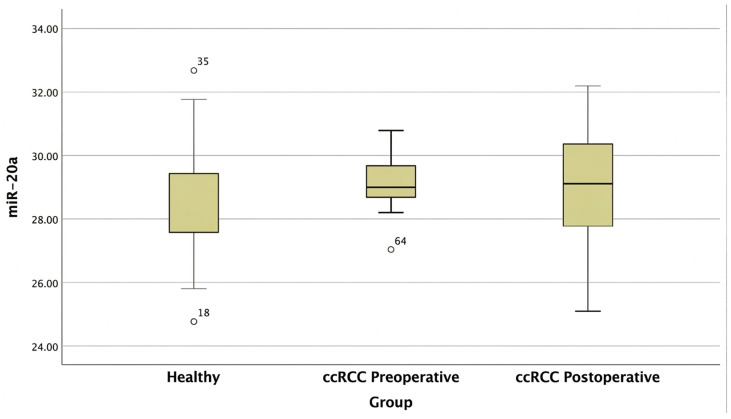
Boxplot showing the expression levels of *miR-20a* across three different clinical groups: healthy controls, ccRCC patients, and patients 5 days post-nephrectomy.

The boxplot (Figure 4) shows that *miR-20a* expression was relatively stable across all groups, with no significant fluctuations, supporting its potential role as an endogenous control in urinary miRNA studies for ccRCC. The outliers are indicated by circles and were present only in healthy individuals and pre-surgery samples. After confirming that the *miR-20a* expression was normally distributed, one-way ANOVA was conducted to compare *miR-20a* expression across the three clinical groups (Table 4).

Post hoc analysis was performed to explore potential pairwise differences in *miR-20a* expression between the clinical groups. When both Tukey’s HSD and Bonferroni adjustments were used for multiple comparisons, no statistically significant differences emerged among the three groups. The coefficient of variation (CV) was 4.98% (SD = 1.42; mean = 28.05) in the healthy controls, 2.71% in the ccRCC patients (SD = 0.79; mean = 29.17), and 6.38% in the postoperative group (SD = 1.85; mean = 28.96.) (Table 5).

### 2.3. Correlation of miR-20a Expression with Pathological, Histological Tumor Variables, and Days of Storage

To investigate the association between *miR-20a* expression and tumor pathological characteristics, analyses were performed focusing on pathological T stage and histological grade (WHO/ISUP) in the ccRCC preoperative samples.

Comparisons of *miR-20a* expression between the T1 group (pT1a and pT1b) and T2 group (pT2a and pT2b) were performed using an independent samples Student’s *t*-test. To further explore whether *miR-20a* expression exhibited a progressive change with increasing tumor stage (from pT1a to pT2b), a trend analysis was conducted using Spearman’s rank correlation. The association between *miR-20a* expression and histological grade was evaluated using Spearman’s rank correlation analysis.

The correlation between miR-20a expression and histological grade groups (low grade: G1–G2; high grade: G3–G4) was assessed using Spearman’s rank correlation analysis. Finally, we analyzed the correlation between miR-20a Ct values and the number of days from urine collection to RNA extraction by Spearman rank test.

All the results of the above-described statistical tests are listed in Table 6.

## 3. Discussion

Urine represents an important source of minimally invasive biomarkers for the diagnosis and prognosis of various diseases including cancer. However, intrinsic variability related to urine concentration, diuretic fluctuations, and numerous physiological and environmental factors—such as circadian rhythm, diet, renal function, and inflammatory or metabolic status—can significantly bias its molecular content, affecting data interpretation. Therefore, optimal standardization of the pre-analytical phase and the adoption of robust normalization strategies during data analysis are crucial steps to correct both the technical and biological variability introduced during sample collection, RNA isolation, reverse transcription, and amplification [13]. A suitable normalizer should display stable expression across all clinical conditions and sample processing variables [15]. Several normalization strategies have been proposed, including global mean normalization, small non-coding RNAs (snoRNAs, snRNAs, rRNAs), and exogenous spike-ins; however, global mean normalization is unsuitable for targeted clinical studies, while alternative controls are biologically dissimilar to miRNAs or account only for technical variability, resulting in suboptimal performance, particularly in extracellular fluids [16].

No universally accepted reference miRNA exists for urinary studies, particularly in kidney cancer, due to the lack of stability in cancer patients [17]. Among the potential endogenous references, *miR-20a*, which belongs to the *miR-17-92* cluster, has been proposed as a candidate reference miRNA in previous cancer-related studies [13,18], but its performance in the urine of ccRCC patients remains insufficiently explored.

J. Oto et al. (2021) systematically evaluated multiple candidate reference miRNAs in urine samples from RCC patients and identified *miR-20a* as a promising urinary normalizer for miRNA quantification in ccRCC patients [13]. To date, this study remains the only available report in the literature specifically addressing the discovery and application of *miR-20a* as an endogenous urinary normalizer in the context of liquid biopsy development for ccRCC.

Building on these findings, the present study sought to further strengthen the evidence supporting miR-20a as a reliable endogenous normalizer in ccRCC, with a particular focus on assessing its stability in stabilized urine samples, rather than in fresh specimens as previously reported. To overcome this issue, we systematically evaluated the expression stability of six candidate urinary miRNAs (miR-15b, miR-16, miR-15a, miR-210-3p, miR-let-7b, and miR-20a-5p) across 99 urine samples collected from a cohort of 77 individuals, including healthy controls, ccRCC patients before surgery, and the same patients 5 days after nephrectomy. The present study cohort showed well-balanced demographic characteristics across the three clinical groups, providing an excellent dataset for evaluating the stability of different miRNAs. The expression stability of the six miRNAs was assessed using RefFinder [19], an integrative web-based tool that combines four widely used algorithms—GeNorm, NormFinder, BestKeeper, and the ΔCt method—by calculating the geometric mean of the stability ranks generated by each approach [15,16,17,18,19,20,21,22]. Based on the overall ranking generated by RefFinder, miR-20a consistently emerged as one of the most stable reference miRNAs across all analytical approaches with a GM of 1778. Notably, *miR-210* consistently showed the poorest stability scores across all four, a finding that is biologically plausible in light of its well-established oncological role in the hypoxia-inducible pathways [11]. In terms of absolute expression, *miR-20a* was uniformly detected across all urine samples, fulfilling a key requirement for a reliable endogenous normalizer in clinical settings [10]. RT-qPCR analysis confirmed these findings, showing similar miR-20a expression across all groups, with comparable Ct values in healthy controls, preoperative, and postoperative ccRCC samples. The substantial overlap of the 95% confidence intervals among groups supports the absence of relevant differences in miR-20a expression. After confirming the normal distribution of the *miR-20a* expression via the Shapiro–Wilk test, one-way ANOVA revealed no statistically significant differences between the groups. In addition, post hoc pairwise comparisons via Tukey’s HSD and Bonferroni correction confirmed the absence of significant differences. Post hoc analyses showed no significant differences in ΔCt values among groups: comparisons between the healthy controls and preoperative, healthy and postoperative, and preoperative and postoperative samples all revealed small mean differences that did not reach statistical significance with either Tukey or Bonferroni correction. The stability of miR-20a expression was further supported by the coefficient of variation (CV) analysis. After confirming the overall stability of miR-20a across the main clinical groups, we further assessed whether its expression remained consistent in patients with tumors characterized by different staging and histological grading.

Student’s *t*-test and Spearman’s rank tests revealed no significant difference or correlations between *miR-20a* expression levels and tumor stage, with all *p* values > 0.05, further supporting its robustness and suitability as an endogenous normalizer independent of disease severity. Spearman’s rank correlation between miR-20a expression and histological grading revealed no significant correlation, indicating that miR-20a expression is not associated with tumor grade in this cohort.

Nevertheless, these results should be interpreted with caution, as they are limited by the relatively small sample size and by the lack of representation of patients with renal tumors beyond the pT2 stage.

The results obtained from the present study are in line with previous findings, indicating that *miR-20a* represents a highly stable endogenous normalizer for urinary miRNA analysis in ccRCC, capable of minimizing technical and biological variability to improve the accuracy and reproducibility of urinary miRNA quantification for potential clinical applications in ccRCC [23]. However, the findings reported by Oto relied on freshly processed urine samples without the use of any stabilizing agents, thereby limiting sample transportability and storage. We standardized the pre-analytical phase by using a urine stabilizing solution, previously employed by other authors for miRNA evaluation [24]. In contrast, the present study investigated the stability of *miR-20a* in urine samples collected and preserved using a dedicated stabilizing solution, which prevents RNA degradation and allows for storage and transport at room temperature for extended periods—even up to two years—without compromising RNA integrity. This preservative ensures uniform protection of small RNAs across different miRNA species, minimizing pre-analytical variability and supporting accurate and reproducible quantification. By maintaining RNA stability during delayed processing, the use of such stabilizers facilitates the collection of samples from centers distant from the analytical laboratory and enhances the feasibility of large-scale, multicenter urinary liquid biopsy studies. Moreover, our analysis demonstrated that the duration of urine storage before RNA extraction did not significantly affect *miR-20a* expression. Despite the wide differences in storage duration among sample groups, Spearman correlation analysis showed no significant association between miR-20a Ct values and storage time, indicating that miR-20a expression was not influenced by storage duration. This step represents an important advancement, ensuring sample integrity and further supporting the robustness of *miR-20a* as an endogenous normalizer for urinary miRNA analyses. Importantly, this approach opens future perspectives for the development of urinary liquid biopsy workflows even in centers distant from the analytical laboratory: urine samples can be collected and stored at room temperature without immediate processing, greatly facilitating sample handling and transport while maintaining reliable miRNA measurements.

Despite these strengths, our study is limited by its relatively small sample and by the restricted multicenter design, as most of the urine samples were collected at a single center. In addition, tumors with stage > pT2 and metastatic disease were not included, and evaluating the behavior of miR-20a in more advanced and metastatic ccRCC would be of particular interest. Additional validation in larger and multicenter cohorts would be valuable to confirm these findings and to further assess the performance of *miR-20a* in comparison with other potential endogenous reference miRNAs. Nonetheless, this investigation provides important evidence that *miR-20a* is a biologically relevant and technically robust endogenous normalizer for urinary miRNA analysis in ccRCC. Its application could help reduce interstudy variability and improve the reproducibility and translational value of urinary miRNA-based liquid biopsy approaches for potential ccRCC diagnosis [23].

## 4. Materials and Methods

### 4.1. Local Ethics Committee

The research was approved by the local ethics committee (protocol no. 3193/18), and informed written consent was obtained from all participants. All procedures and techniques adhered to relevant guidelines as well as national and international regulations. Urine sample collection was conducted following legal and ethical standards. All the data and urine samples collected from the participants were evaluated anonymously.

### 4.2. Patient, MicroRNA Selection, Samples Collection

The study included a total of 77 individuals, comprising 47 healthy controls and 30 patients diagnosed with ccRCC. Overall, 99 urine samples were collected: 47 samples from healthy controls, 30 urine samples from ccRCC patient preoperative, and 22 postoperative urine samples collected five days after nephrectomy from the same patients. Fourteen individuals were recruited at the University of Chieti–Pescara, including 8 healthy controls and 6 ccRCC patients, who provided a total of 16 urine samples (8 from healthy controls, 6 preoperative samples, and 2 postoperative samples). The remaining participants were enrolled at the Urology Clinic of the University of Perugia. To ensure that the study population was as homogeneous as possible, we chose to include only the ccRCC subtype, which is also the most prevalent histological variant of renal cancer [2]. We included all patients aged over 30 years with renal masses, while individuals with urinary infections, renal lithiasis, or those affected by other cancers were excluded. For healthy controls, exclusion criteria included a history of urinary tract infections, renal lithiasis, or neoplastic diseases. The expression of six candidate miRNAs (*miR-15b*, *miR-16*, *miR-15a*, *miR-210-3p*, *miR-let-7b*, and *miR-20a-5p*) was quantified by RT-qPCR in urine samples. *MiR-20a* was selected based on previous evidence reported by J. Oto et al. (2021) [13]. The remaining five miRNAs were selected according to our previous studies, in which they were identified through bioinformatic analyses and experimentally validated in renal cancer-related models [25]. Stability ranking was performed via the online tool called RefFinder (https://www.ciidirsinaloa.com.mx/RefFinder-master/?type=reference, accessed on 20 January 2025) [14]. RefFinder is a web-based tool designed to assess and rank candidate reference genes comprehensively via large experimental datasets. This platform integrates the results from four well-established computational algorithms commonly used for reference miRNA validation: geNorm, which evaluates miRNA stability on the basis of pairwise variation; normFinder, which estimates both intra- and intergroup variability; BestKeeper, which assesses stability on the basis of standard deviation and coefficient of variation of Ct values; and the comparative ΔCt method, which examines relative expression differences between pairs of miRNAs across samples [19,20,21,22]. Each method provides an independent ranking of miRNA stability, and RefFinder combines these individual outputs to generate a comprehensive final ranking by calculating the geometric mean (GM) of the individual miRNA weights assigned by each algorithm [14].

To investigate the association between *miR-20a* expression and tumor pathological characteristics, pathological staging was defined according to the European Association of Urology (EAU) Guidelines (2025). To improve statistical robustness, given the limited number of cases with higher stages (>pT1b), tumors were dichotomized into two groups:

T1: pT1a and pT1b;

T2: pT2a and pT2b.

No tumors beyond pT2 were included, as such stages were not represented in the cohort.

Similarly tumors were categorized according to the WHO/ISUP grading system into:

Low grade: G1–G2;

High grade: G3–G4.

Urine samples were collected from both patients and HSs in sterile 50 mL containers during the afternoon. Specifically, patient urine samples were obtained following hospital admission (approximately 2:00 PM) on the day before surgery. Urine stabilization occurred within 4 h of collection with 1 mL of “Urine Preservative Single Dose Ampules” (Norgen Biotek, Thorold, ON, Canada), preserving genetic material for up to 2 years at room temperature (20–25 °C).

### 4.3. Total RNA Extraction and Reverse Transcription

Total RNA was extracted from 3 mL of urine via the QIAamp Circulating Nucleic Acid Kit (Qiagen ©, Hulsterweg 82, 5912 PL Venlo, The Netherlands). Before the RNA isolation procedure was started, 1 μL of *UniSp2* and *UniSp4* RNA spike-in mixture (Qiagen ©, Hulsterweg 82, 5912 PL Venlo, The Netherlands) was added to each sample to evaluate the efficiency and reproducibility of the extraction procedure. Urinary RNA was reverse transcribed (RT) via the miRCURY LNA RT Kit (Qiagen ©, Hulsterweg 82, 5912 PL Venlo, The Netherlands), which includes *UniSp6*, a proprietary synthetic RNA spike-in template (Qiagen ©, Hulsterweg 82, 5912 PL Venlo, The Netherlands) for monitoring performance. The RT was performed following the manufacturer’s protocol, which includes urine centrifugation in order to obtain cell-free urine and was carried out under the following cycling conditions: initial incubation at 42 °C for 60 min, followed by another incubation at 95 °C for 5 min to inactivate the RT enzyme. The resulting cDNA was stored at −20 °C following the manufacturer’s instructions, and it was thawed and diluted 30-fold with sterile water as soon as it was needed for subsequent analyses.

### 4.4. Quantitative PCR Analysis

For each sample, three internal controls were amplified: *UniSP2* and *UniSP4* (Qiagen spike-in control for miRNA recovery assessment) and *UniSP6* (spike-in control used to evaluate reverse transcription efficiency). The expression of six target urinary miRNAs (*miR-15b*, *miR-16*, *miR-15a*, *miR-210-3p*, *miR-let-7b*, and *miR-20a-5p*) was analyzed via RT-qPCR in triplicate, with three internal controls and a no-template negative control (NTC) in duplicate. *UniSP2* and *UniSP4* (Qiagen spike-in control for miRNA recovery assessment) as well as *UniSP6* (spike-in control used to evaluate reverse transcription efficiency) were used as internal controls. RT-qPCR was performed via a Rotor-Gene Q (Qiagen ©, Hulsterweg 82, 5912 PL Venlo, The Netherlands) instrument and the miRCURY LNA SYBR^®^ Green PCR Kit (Qiagen ©, Hulsterweg 82, 5912 PL Venlo, The Netherlands). The manufacturer’s protocol included the following cycling conditions: initial denaturation at 95 °C for 2 min, followed by 40 cycles of 95 °C for 10 s, 56 °C for 60 s for denaturation and annealing/extension phases, and a ramp from 60 to 95 °C for melting curve analysis. miRNA quantification was performed via the cycle threshold (Ct) method.

### 4.5. Statistical Analysis

Raw Ct values with a mean <35 obtained via RT-qPCR were considered valuable outputs and were analyzed via IBM SPSS Statistics software (version 29.0.2.0 (20), IBM Corp., Armonk, NY, USA). Graphs and statistical plots were created via IBM SPSS Statistics software (version 29.0.2.2, IBM Corp., Armonk, NY, USA).

A Pearson chi-square test of independence was conducted to examine sex distribution across the three clinical groups. The normality of the age distribution was assessed via the Shapiro–Wilk test. Differences in age across the three clinical groups (healthy subjects, ccRCC patients preoperative, and 5 day postoperative patients) were assessed via the Kruskal–Wallis test because of the non-normal distribution in only one group (healthy control).

To select the best endogenous miRNA for normalization, analyses via the comprehensive online tool RefFinder were conducted on six different candidate miRNAs as reference endogenous miRNAs [14]. The normality of the top-ranked miRNA expression data was assessed via the Shapiro–Wilk test (Table 2). Differences in miRNA expression levels between groups (healthy controls, patients at diagnosis, and 5 days post-surgical follow-up) were analyzed via one-way ANOVA followed by Tukey’s and Bonferroni post hoc test or independent-sample *t* tests when two groups were compared (Table 3 and Table 4, respectively). In cases where the data did not meet the normality assumptions, nonparametric equivalents were used. Coefficients of variation (CVs) were calculated to evaluate the intragroup expression stability of the selected miRNAs, with a CV less than 50% considered indicative of stable expression.

## 5. Conclusions

In summary, the present study supports the use of *miR-20a* as a stable and reliable endogenous normalizer for quantifying urinary miRNAs in patients affected by ccRCC. Importantly, our data show that *miR-20a* expression was not affected by the duration of urine storage prior to RNA extraction, even when samples were stored for extended periods, highlighting its robustness under varying pre-analytical conditions.

Notably, by standardizing the pre-analytical phase with a urine stabilizing solution previously used in other miRNA studies, we ensured sample integrity, representing a significant step forward in the reliable analysis of urinary miRNAs. This approach opens future perspectives for urinary liquid biopsy applications, even in collection centers distant from analytical laboratories, as urine samples can be safely stored at room temperature and processed later without compromising data quality. The use of *miR-20a* as a reference control may thus contribute to minimizing inter-study variability, improving assay reproducibility, and enhancing the translational applicability of urinary miRNA-based liquid biopsy approaches for kidney cancer diagnostics. Further large-scale validation studies are needed to confirm these results and to support the clinical implementation of miR-20a normalization strategies in future biomarker discovery and validation studies. In particular, it would be interesting to assess whether this normalizer retains its robustness in metastatic disease and across renal tumor histology other than ccRCC.

## Figures and Tables

**Table 1:** Demographic characteristics of the study population across the three clinical groups. No significant differences were found in sex distribution or age.

		HSs (*n* = 47)	ccRCC Preoperative (*n* = 30)	ccRCC Postoperative (*n* = 22)	Kruskal–Wallis Test	Pearson Chi-Square
Sex	Male	22	19	14	Not Applicable	χ^2^ (2) = 2.773 *p* = 0.250
	Female	25	11	8		
Age	Mean ± S.D. ^1^	64.91 ± 10.67	64.63 ± 12.31	63.72 ± 12.57	H(2): 0.064. *p* = 0.969	Not Applicable
	Range	51–93	40–86	40–84		
Passed SW ^2^ Test		No (*p* ≤ 0.001)	Yes (*p* = 0.31)	Yes (*p* = 0.42)		
Days from collection to extraction	Median	21.8	107.7	92.9		
	IQR ^3^	0–9 (9)	54–119 (67)	43–119 (78)		

^1^ Standard deviation. ^2^ Shapiro–Wilk test. ^3^ Interquartile range.

**Table 2:** Descriptive statistics and distribution analysis of the top-ranked urinary *miR-20a* Ct by clinical group.

Clinical Group	N	Mean	Standard Deviation	Standard Error	95% CI for the Mean		Min	Max
					Lower Bound	Upper Bound		
Healthy	47	28.50	1.42	0.21	28.08	28.92	24.79	32.68
ccRCC Preoperative	30	29.17	0.79	0.144	28.87	29.47	27.04	30.77
ccRCC Postoperative	22	28.96	1.85	0.39	28.14	29.78	25.09	32.14
Total	99	28.87	1.35	0.25	28.36	29.39	25.47	31.86

**Table 3:** Normality tests for urinary *miR-20a* Ct expression in each clinical group.

	Clinical Group	Shapiro–Wilk		
		Statistic	df	*p*-Value
*miR-20a* expression	Healthy	0.963	47	0.140
	ccRCC Preoperative	0.959	30	0.288
	ccRCC Postoperative	0.983	22	0.954

**Table 4:** One-way ANOVA for urinary *miR-20a* Ct expression across clinical groups.

Source	Sum of Squares	df	MeanSquare	F	*p*-Value
Between groups	8.883	2	4.441	2.324	0.103
Within groups	183.436	96	1.911		
Total	192.319	98			

**Table 5:** Post hoc multiple comparisons of *miR-20a* expression levels across clinical groups. Pairwise differences were assessed via both Tukey’s HSD and the Bonferroni correction.

	Dependent Variable: *miR-20a* Expression						
	(I) Clinical Group	(J) Clinical Group	Mean Difference (I-J)	Std. Error	Sig.	95% Confidence Interval	
						Lower Bound	Upper Bound
Tukey HSD	Healthy	Preoperative	−0.668	0.32307172	0.102	−1.438	0.100
		Postoperative	−0.462	0.357	0.401	−1.313	0.388
	ccRCC Preoperative	Healthy	0.669	0.323	0.102	−0.101	144
		Postoperative	0.206	0.388	0.856	−0.718	1.129
	ccRCC Postoperative	Healthy	0.463	0.357	0.401	−0.388	1.313
		Preoperative	−0.206	0.388	0.856	−1.129	0.718
Bonferroni	Healthy	Preoperative	−0.668	0.323	0.124	−1.456	0.119
		Postoperative	−0.463	0.357	0.595	−1.333	0.2408
	ccRCC Preoperative	Healthy	0.669	0.323	0.124	−0.119	1.456
		Postoperative	0.206	0.388	1.000	−0.739	1.151
	ccRCC Postoperative	Healthy	0.463	0.357	0.595	−0.408	1.332
		Preoperative	−0.206	0.0388	1.000	−1.151	0.739

**Table 6:** Statistical analyses of *miR-20a* expression and tumor staging, grading, and day of storage. Student’s *t*-test (**A**) and Spearman’s rank correlation analysis (**B**) assessed the correlation between *miR-20a* expression and tumor staging groups. Spearman’s rank correlation analysis (**C**) between *miRNA-20a* expression and tumor grading. Spearman correlation (**D**) between *miR-20a* Ct values and days from urine collection to RNA extraction. In the table, *t* indicates the *t*-value, df the degrees of freedom, and *p* the *p*-value.

**A**	Student’s *t*-test	*miR-20a* expression	*t*	df	*p*	Mean Difference	Standard Error
			−1.625	22	0.118	−0.669	0.412
						*miR20a* expression	Tumor Staging
**B**	Spearman’s rho	*miR-20a* expression	Correlation Coefficient			1.000	0.114
			Sig. (2-tailed)				0.547
			N			30	30
		Tumor Staging	Correlation Coefficient			0.114	1.000
			Sig. (2-tailed)			0.547	
			N			30	30
						*miR-20a* expression	Tumor Grading
**C**	Spearman’s rho	*miRNA-20a* expression	Correlation Coefficient			1000	0.153
			Sig. (2-tailed)				0.420
			N			30	30
		Tumor Grading	Correlation Coefficient			0.153	1000
			Sig. (2-tailed)			0.420	
			N			30	30
						*miR-20a* expression	Days from collection to extraction
**D**	Spearman’s rho	*miRNA-20a* expression	Correlation Coefficient			1.000	0.167
			Sig. (2-tailed)				0.100
			N			99	99
		Days from collection to extraction	Correlation Coefficient			0.167	1.000
			Sig. (2-tailed)			0.100	
			N			99	99

## Data Availability

The datasets generated and analyzed during the current study are available from the corresponding author upon reasonable request.

## Supplementary Materials

The following supporting information can be downloaded at: https://www.mdpi.com/article/10.3390/ijms27073323/s1.
