# Supplementary material for: Urinary miRNA Analysis for Clear Cell Renal Cell Carcinoma: miR-20a as a Key Endogenous Normalizer
Source: Int J Mol Sci. 2026 Apr 7;27(7):3323. doi: 10.3390/ijms27073323 (PMC13073742; doi:10.3390/ijms27073323)
Supplement: Supplementary file 1 [file ijms-27-03323-s001.zip › ijms-4149443-supplementary.pdf]

# Supplementary Materials

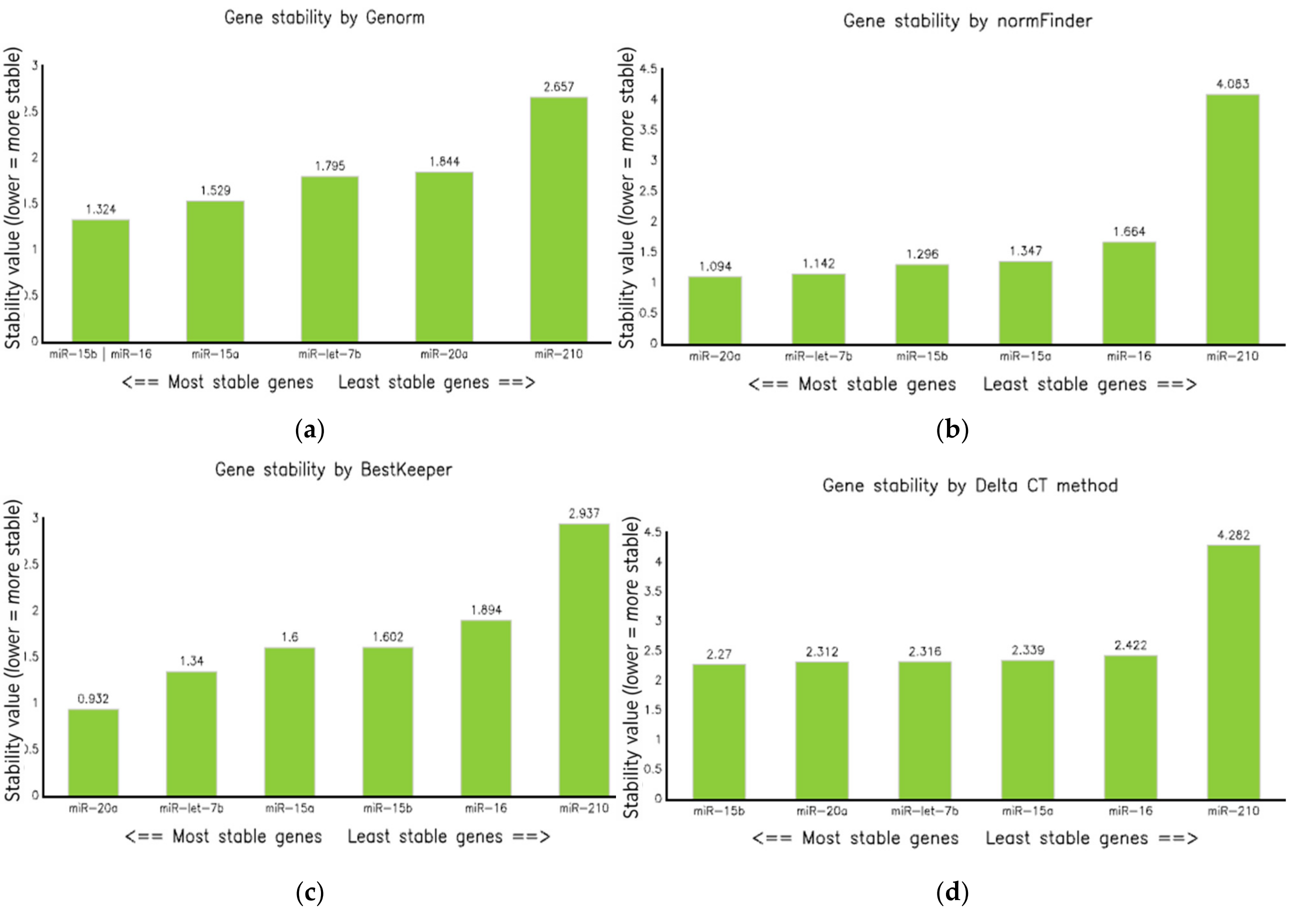


**Figure S1.** miRNA stability analysis via: (**a**) the Genorm algorithm; (**b**) normFinder algorithm; (**c**) BestKeeper algorithm and (**d**) Delta Ct method.

### miRNA PCR Assays

The following miRNA-specific assays were used for RT–qPCR analyses:

- hsa-miR-20a-5p, miRCURY LNA miRNA PCR Assay (GeneGlobe ID: YP00204292)
- hsa-miR-16-5p, miRCURY LNA miRNA PCR Assay (GeneGlobe ID: YP00205702)
- hsa-miR-15b-5p, miRCURY LNA miRNA PCR Assay (GeneGlobe ID: YP00204243)
- hsa-miR-210-3p, miRCURY LNA miRNA PCR Assay (GeneGlobe ID: YP00204333)
- hsa-let-7b-5p, miRCURY LNA miRNA PCR Assay (GeneGlobe ID: YP00204750)
- hsa-miR-15a-5p, miRCURY LNA miRNA PCR Assay (GeneGlobe ID: YP00204066)

All miRNA primer assays were purchased from Qiagen and shared the same catalog number (**Cat. No. 339306**).

### Kits and Reagents

- **miRCURY LNA RT Kit** for reverse transcription (Qiagen; **Cat. No. 339340**)

- **miRCURY LNA SYBR Green PCR Kit (200 reactions)** (Qiagen; **Cat. No. 339345**)
- **QIAamp Circulating Nucleic Acid Kit (50)** for miRNA extraction (Qiagen; **Cat. No. 55114**)
